# 2-Butoxyethanol: MAK-Begründung, Nachtrag

**DOI:** 10.34865/mb11176d11_2ad

**Published:** 2026-06-30

**Authors:** Andrea Hartwig, MAK Commission

**Affiliations:** 1 Institut für Angewandte Biowissenschaften, Abteilung Lebensmittelchemie und Toxikologie, Karlsruher Institut für Technologie (KIT) Adenauerring 20a, Geb. 50.41 76131 Karlsruhe Deutschland; 2 Ständige Senatskommission zur Prüfung gesundheitsschädlicher Arbeitsstoffe, Deutsche Forschungsgemeinschaft, Kennedyallee 40, 53175 Bonn, Deutschland. Weitere Informationen: Ständige Senatskommission zur Prüfung gesundheitsschädlicher Arbeitsstoffe | DFG

**Keywords:** 2-Butoxyethanol, Entwicklungstoxizität, erhöhtes Atemvolumen, Reizwirkung, Hämatotoxizität

## Abstract

The German Senate Commission for the Investigation of Health Hazards of Chemical Compounds in the Work Area (MAK Commission) re-evaluated the assignment of 2-butoxy­ethanol [111-76-2] to Pregnancy Risk Group C (“Damage to the embryo or foetus is unlikely if the MAK value or the BAT value is observed.”). Considering the increased respiratory volume at the workplace (see List of MAK and BAT Values, Section I b and I c), the NOAECs for developmental toxicity for rats and rabbits are 2.5 and 5 times the MAK value of 10 ml/m^3^, respectively. In rabbits, a direct developmental toxic effect is not to be assumed due to the predominant maternal toxicity. For rats, it is plausible to assume that the developmental toxicity is almost exclusively due to the haemolysis caused by the metabolite butoxyacetic acid. Humans have been shown to be significantly less sensitive towards this effect, by a factor of 16. This results in a sufficient 40-fold (2.5 × 16) margin between the NOAEC for developmental toxicity and the MAK value. Therefore, the assignment to Pregnancy Risk Group C has been confirmed.

**Table:** 

**MAK-Wert (2006)**	**10 ml/m^3^ (ppm) ≙ 49 mg/m^3^^a)^**
**Spitzenbegrenzung (2006)**	**Kategorie I, Überschreitungsfaktor 2**
	
**Hautresorption (1980)**	**H**
**Sensibilisierende Wirkung**	**–**
**Krebserzeugende Wirkung**	**–**
**Fruchtschädigende Wirkung (1985)**	**Gruppe C**
**Keimzellmutagene Wirkung**	**–**
	
**BAT-Wert (2015)**	**150 mg Butoxyessigsäure (nach Hydrolyse)/g Kreatinin**
CAS-Nr.	111-76-2
**1 ml/m^3^ (ppm) ≙ 4,903 mg/m^3^**	**1 mg/m^3^ ≙ 0,204 ml/m^3^ (ppm)**

^a)^ MAK-Wert für die Summe der Luftkonzentrationen von 2-Butoxyethanol und 2-Butoxyethylacetat

Zu 2-Butoxyethanol liegen eine Begründung (Henschler 1983), ein Nachtrag zur Spitzenbegrenzung (Greim 2001), eine Gesamtreevaluierung (Greim 2007) sowie eine Reevaluierung der kanzerogenen Wirkung (Hartwig und MAK Commission^2018^) vor.

Seit dem Jahr 2016 berücksichtigt die Kommission bei Stoffen, deren MAK-Wert auf systemischen Effekten basiert und aus inhalativen Tierversuchen oder Probandenstudien in Ruhe abgeleitet wurde, dass das Atemvolumen am Arbeitsplatz höher als unter diesen experimentellen Bedingungen ist. Dies gilt jedoch nicht für Gase und Dämpfe, wenn deren Blut:Luft-Verteilungskoeffizient < 5 ist (DFG 2025, Abschnitt I b und I c; Hartwig und MAK Commission^2017^). Der Blut:Luft-Verteilungskoeffizient liegt für 2-Butoxyethanol bei 7965 (Meulenberg und Vijverberg 2000). Daher muss das erhöhte Atemvolumen berücksichtigt werden.

In diesem Nachtrag wird die Zuordnung zur Schwangerschaftsgruppe reevaluiert, da bisher das erhöhte Atemvolumen nicht berücksichtigt worden ist. Zudem werden bisher nicht zitierte Studien ergänzt.

## Toxikokinetik und Metabolismus

### Mensch

2-Butoxyethanol wird sowohl über die Atemwege als auch über die Haut sehr gut resorbiert (Hartwig und MAK Commission^2018^).

Im Nachtrag von 2018 (Hartwig und MAK Commission^2018^) wird ausführlich dargelegt, dass die dermale Resorption von 2-Butoxyethanol aus der Gasphase nicht, wie von Johanson und Boman (1991) publiziert, 75 % der gesamten Aufnahme beträgt. In dieser Studie wurde das Kapillarblut zugrunde gelegt, jedoch ist die Butoxyessigsäurekonzentration im venösen Blut ein geeigneteres Maß für die systemische Belastung. Der tatsächliche Beitrag der dermalen Resorption an der gesamten Aufnahme bei Ganzkörperexposition liegt bei etwa 15 % bis 27 %; bei körperlicher Tätigkeit bei 5 % bis 9 % (Corley et al. 1997). Eine weitere Studie bestätigt dies: die dermale Resorption aus der Gasphase betrug unter Ruhebedingungen und leichter Bekleidung bei 25 °C und 40 % Luftfeuchtigkeit 11 % der gesamten Belastung. Beim Tragen von Overalls bei 30 °C und 60 % Luftfeuchtigkeit stieg der Anteil auf 39 % (Jones et al. 2003).

Beim Menschen wird der überwiegende Teil des in den Organismus aufgenommenen 2-Butoxyethanols oxidativ zu Butoxyessigsäure metabolisiert, die entweder in freier Form oder als Glutaminkonjugat (N-Butoxyacetylglutamin) sowie in geringen Mengen auch als Glycinkonjugat im Urin ausgeschieden wird. Der Anteil der in konjugierter Form ausgeschiedenen Butoxyessigsäure an der Gesamtausscheidung variiert sehr stark. Glucuronide oder Sulfate als Konjugate sind beim Menschen nicht nachgewiesen worden (Hartwig und MAK Commission^2018^).

Zur Ausscheidung der Butoxyessigsäure mit dem Urin liegen nur sehr wenige Daten vor. Eine Probandenstudie an zwei Frauen und zwei Männern mit zweistündiger Ganzkörperexposition (inhalativ und dermal) (Jones und Cocker 2003) sowie eine Arbeitsplatzstudie an 15 weiblichen und 37 männlichen Siebdruckern (Laitinen et al. 1998) lässt nicht auf einen geschlechtsabhängigen Unterschied bei der renalen Ausscheidung der Butoxyessigsäure schließen.

### Tier

Bei der männlichen Ratte beträgt der Prozentsatz der dermalen Aufnahme aus der Gasphase etwa 15 % an der Gesamtaufnahme nach Ganzkörperexposition (Lee et al. 1998 a).

Bei Ratten wurden im Urin neben der freien Butoxyessigsäure im Gegensatz zum Menschen das Glucuronid- und Sulfatkonjugat nachgewiesen, jedoch kein Glutaminkonjugat (Hartwig und MAK Commission^2018^).

Toxikokinetische Daten an trächtigen Tieren liegen nicht vor. Daher werden Daten zu weiblichen Tieren herangezogen.

Im Rahmen einer bereits im Nachtrag von 2007 beschriebenen 2-Jahre-Studie des NTP an männlichen und weiblichen F344/N-Ratten und männlichen und weiblichen B6C3F1-Mäusen mit Ganzkörperexposition (6 Stunden/Tag, 5 Tage/Woche) (Greim 2007; NTP 2000) wurden toxikokinetische Untersuchungen vorgenommen. Die analytischen Konzentrationen lagen bei 0; 31,2; 62,5 und 125 ml 2-Butoxyethanol/m^3^ für Ratten und bei 0; 62,5; 125 und 248 ml/m^3^ für Mäuse. Postexpositionsblutproben wurden einen Tag, zwei Wochen sowie 3, 6, 12 und 18 Monate nach Expositionsbeginn genommen (jeweils mehrere Zeitpunkte nach der 6-Stunden-Exposition, für die Zeitpunkte einschließlich sechs Monate: nach 10, 20, 40, 180, 360 und 720 Minuten). Postexpositions-16-h-Urinproben (Tiere einzeln in Stoffwechselkäfigen, während die Urinproben 16 h auf Eis gesammelt wurden) wurden nach zwei Wochen sowie nach 3, 6, 12 und 18 Monaten gesammelt. Es wurde gezeigt, dass systemisch aufgenommenes 2-Butoxyethanol unabhängig von der Expositionskonzentration schnell aus dem Blut eliminiert wird (Halbwertszeiten: Ratte: < 10 min, Maus: < 5 min, jeweils nach eintägiger Exposition). Eine proportionale Zunahme der AUC (Area Under the Curve) von 2-Butoxyethanol im Blut relativ zur Zunahme der Expositionskonzentration weist auf eine lineare Kinetik von 2-Butoxyethanol hin. Im Gegensatz dazu nahm die Butoxyessigsäure-Eliminationsrate aus dem Blut mit zunehmender Expositionskonzentration einer nichtlinearen Kinetik folgend ab. Mäuse eliminierten 2-Butoxyethanol und Butoxyessigsäure schneller aus dem Blut als Ratten. Geschlechtsbezogene Unterschiede bei der Butoxyessigsäure-Elimination waren bei Ratten deutlicher ausgeprägt: Bei weiblichen Ratten war die Elimination von Butoxyessigsäure aus dem Blut geringer als bei männlichen Ratten. So lag die 16-h-Postexpositionsurinausscheidung von Butoxyessigsäure bei männlichen Ratten etwa doppelt so hoch im Vergleich zu weiblichen Ratten. Die unterschiedliche Ausscheidung von Butoxyessigsäure über die Nieren bei männlichen und weiblichen Ratten ist wahrscheinlich für den geschlechtsabhängigen Unterschied der Blutprofile verantwortlich (Dill et al. 1998). Für die geringere renale Elimination der Butoxyessigsäure bei weiblichen im Vergleich zu männlichen Ratten sind vermutlich die organischen Anionentransporter OAT1 (Organic Anion Transporter), OAT3, URAT1 (Urate/Anion Exchanger) verantwortlich. Die Genexpression sowie die Proteingehalte dieser Transporter sind in den Nieren bei männlichen Ratten höher als bei weiblichen Ratten (Burckhardt 2012; Sekine et al. 2006). Die Autoren der 2-Jahre-Studie folgerten, dass die Eliminationskinetik von 2-Butoxyethanol und Butoxyessigsäure nach wiederholter inhalativer Gabe von 2-Butoxyethanol abhängig ist von der Spezies, dem Geschlecht, dem Alter der Tiere bei Exposition, der Expositionszeit (bedingt durch physiologische oder biochemische Veränderungen, die mit dem Alter auftreten) und der Expositionskonzentration (Dill et al. 1998).

Die normalisierte (auf Kreatinin bezogene) Ausscheidung von Butoxyessigsäure mit dem Urin bei weiblichen Ratten nach zwei Wochen (entspricht am ehesten der Gestationsdauer) betrug bei 31,2 ml 2-Butoxyethanol/m^3^ 230 mg Butoxyessigsäure/g Kreatinin, bei 62,5 ml 2-Butoxyethanol/m^3^ 500 mg Butoxyessigsäure/g Kreatinin und bei 125 ml 2-Butoxy­ethanol/m^3^ 1070 mg Butoxyessigsäure/g Kreatinin. Der erhebliche Unterschied bei der 16-h-Postexpositionsurinausscheidung von Butoxyessigsäure zwischen männlichen und weiblichen Ratten wird durch die Normalisierung auf Kreatinin reduziert, vor allem bei der niedrigsten Konzentration (Dill et al. 1998). Die Kreatininkonzentrationen im Urin werden in der Publikation nicht dargestellt. Aus einer weiteren Publikation der Arbeitsgruppe geht hervor, dass bei F344-Ratten die Kreatininkonzentrationen im Urin von männlichen Tieren bei 24 ± 5,1 mg Kreatinin/dl Urin und bei weiblichen Tieren bei 34 ± 17 mg Kreatinin/dl Urin liegen (Lee et al. 1998 b). Dies erklärt die geringeren Abstände der Butoxyessigsäure-Ausscheidung zwischen männlichen und weiblichen Tieren nach Kreatininbezug im Vergleich zur 16-h-Postexpositionsurinausscheidung.

Im Vergleich zum Menschen kommt es also bei der Ratte zu einer schnelleren Bildung von Butoxyessigsäure, höheren Blutkonzentrationen und höherer AUC, jedoch wird beim Menschen der Metabolit langsamer aus dem Blut eliminiert. Dies zeigt sich in einer geringeren Ausscheidung der Butoxyessigsäure mit dem Urin beim Menschen im Vergleich zur Ratte (Corley et al. 1994, 2005; Hartwig und MAK Commission^2018^).

### Vergleich der hämolytischen Wirkstärke bei Mensch und Ratte

Der Metabolit Butoxyessigsäure ist verantwortlich für die hämolytischen Effekte (ECETOC 2005 a, b; NTP 2000), 2-Butoxy­ethanol selbst besitzt keine hämolytische Aktivität (NTP 2000).

Für die hämolytische Wirkung von 2-Butoxyethanol gibt es einen spezies- und geschlechtsspezifischen Unterschied. Ratten und Mäuse sind für derartige Effekte sensitiver, gefolgt von Kaninchen und Primaten. Schweine, Hunde, Katzen, Meerschweinchen und der Mensch sind relativ insensitiv. Für Affen und Ratten ist gezeigt worden, dass weibliche Tiere empfindlicher auf die hämolytischen Effekte durch 2-Butoxyethanol reagieren als männliche Tiere (NTP 2000).

Im Nachtrag von 2018 wird ausführlich auf die unterschiedliche Empfindlichkeit von Mensch und Ratte eingegangen. Aus dem Vergleich der erhöhten osmotischen Fragilität von Erythrozyten bei Probanden (insgesamt sechs Probanden) und Ratten nach 2-Butoxyethanol-Exposition ergibt sich, dass Ratten mindestens 6-mal so empfindlich wie der Mensch sind. Jedoch sind nur wenige Probanden untersucht worden. Nach 4-stündiger Inkubation von Humanblut mit 8 mM Butoxyessigsäure wurde eine geringere Hämolyse verursacht als mit 0,5 mM Butoxyessigsäure in Rattenblut. Damit sind Rattenerythrozyten 16-mal so empfindlich wie Humanerythrozyten. Für Ratten- als auch für Humanblut nimmt die hämolytische Wirkung mit der Expositionszeit zu, sodass eine stärkere Wirkung nach längerer Exposition, wie bei 8-stündiger Exposition am Arbeitsplatz, zu erwarten ist. Am Arbeitsplatz kommt es zu einer langsameren Anflutung und einer langsameren Einstellung des Fließgleichgewichts im Vergleich zum In-vitro-Versuch, der einer Bolus-Applikation ähnlich ist, bei der sofort die maximale Konzentration einwirkt. Insgesamt kann aus den Daten geschlossen werden, dass bezogen auf die Konzentration von Butoxyessigsäure im Blut, Ratten etwa 16-mal so empfindlich sind wie Menschen (Hartwig und MAK Commission^2018^).

Im Nachtrag von 2018 wird erläutert, dass einem PBPK (Physiologisch-basierten pharmakokinetischen)-Modell zufolge die 6-stündige Ganzkörperexposition des Menschen gegen eine gesättigte Dampfatmosphäre von 2-Butoxyethanol von 1160 ml/m^3^ einer Konzentration von 1,5 mM Butoxyessigsäure im Blut entspricht (Corley et al. 1994). Das heißt, die für die Hämolyse von Humanerythrozyten nötige Konzentration von 8 mM Butoxyessigsäure wird nicht erreicht. Ratten weisen unter diesen Bedingungen bei Ganzkörperexposition im Blut etwa 4 mM auf, wobei bei 0,5 mM bereits eine leichte Hämolyse von Rattenerythrozyten zu beobachten ist (Hartwig und MAK Commission^2018^).

## Erfahrungen beim Menschen

### Reproduktionstoxizität

Hierzu liegen keine Daten vor.

## Tierexperimentelle Befunde und In-vitro-Untersuchungen

### Reproduktionstoxizität

#### Fertilität

Im Nachtrag von 2007 wird erwähnt, dass in einer Studie an Swiss-Mäusen mit kontinuierlicher Verpaarung bei den Muttertieren, die 21 Wochen lang mit 1300 mg 2-Butoxyethanol/kg KG und Tag im Trinkwasser behandelt und mit Kontrolltieren verpaart worden waren, die Anzahl der Würfe um 58 % und die Wurfgröße um 66 % geringer war als bei unbehandelten Paaren. Der NOAEL für Fertilität liegt bei 700 mg 2-Butoxyethanol/kg KG und Tag. Bei dieser Dosis war die männliche Fertilität unbeeinträchtigt (Greim 2007; Heindel et al. 1990).

Mehreren Übersichtsartikeln zufolge sind in zahlreichen subakuten, subchronischen und chronischen Studien mit 2-Butoxy­ethanol keine spezifischen Effekte auf die Reproduktionsorgane beobachtet worden (Greim 2007).

Neue Generationenstudien oder Studien zur Fertilität sind seit dem Nachtrag von 2007 nicht durchgeführt worden.

#### Entwicklungstoxizität

Wie im Nachtrag von 2007 im Detail tabellarisch aufgeführt, treten in Entwicklungstoxizitätsstudien an Ratte, Maus oder Kaninchen nach inhalativer bzw. oraler Verabreichung bei maternaltoxischen Konzentrationen entwicklungstoxische Effekte auf. Beobachtet wurden vor allem erhöhte Mortalität und Resorptionen von Feten bzw. Embryonen sowie verzögerte Ossifikation (ECETOC 2005 b; Fastier et al. 2005; Greim 2007). Als NOAEC/NOAEL für Entwicklungstoxizität nach Inhalation lassen sich bei Ratten 50 ml/m^3^ und bei Kaninchen 100 ml/m^3^ (Greim 2007; Tyl et al. 1984) sowie nach oraler Gabe bei Ratten 100 mg/kg KG und Tag (Greim 2007) und bei Mäusen 650 mg/kg KG und Tag (Greim 2007; Wier et al. 1987) ableiten. Die entsprechenden Effektkonzentrationen liegen nach Inhalation bei Ratten bei 100 ml/m^3^ und bei Kaninchen bei 200 ml/m^3^ (Greim 2007; Tyl et al. 1984) sowie nach oraler Gabe bei Ratten bei 200 mg/kg KG und Tag (Greim 2007) und bei Mäusen bei 1000 mg/kg KG und Tag (Greim 2007; Wier et al. 1987). Teratogene Effekte wurden bei Ratten, Mäusen und Kaninchen nicht festgestellt (ECETOC 2005 a, b; Fastier et al. 2005; Greim 2007).

Die Studien mit inhalativer Gabe werden in Tabelle 1 aufgeführt.

Neue Studien liegen nicht vor.

**Tab. 1:** Entwicklungstoxizitätsstudien nach inhalativer Verabreichung von 2-Butoxyethanol mit Konzentrationen bis 200 ml/m^3^

**Spezies, Stamm, Anzahl pro Gruppe**	**Exposition**	**Befunde**	**Literatur**
**Ratte**, F344, 30‑31 ♀	**ähnlich OECD TG 414**, **GD 6–15**, 0, 25, 50, 100, 200 ml/m^3^, Ganzkörperexposition, 6 h/d, Untersuchung GD 21	**50 ml/m^3^: NOAEC Entwicklungs- u. Maternaltoxizität**; **ab 100 ml/m^3^**: Muttertiere: hämatologische Effekte, Hämaturie, Hämoglobinurie, KG-Zunahme GD 6–15 ↓ (24,9 ± 6,0; 24,1 ± 8,0; 20,6 ± 7,9; 17,7 ± 9,2* (28 %); ‑3,8 ± 17,1* g); Feten: nicht ossifizierter Halswirbelkörper Nr 6: Fetenbasis: 2/106 (1,9 %), 4/106 (3,8 %), 5/92 (5,4 %), 11/109 (10,1 %), 12/50 (24,0 %); Wurfbasis: 2/23 (8,7 %), 4/22 (18,2 %), 4/20 (20,0 %), 10/22* (45,5 %), 10/15* (66,7 %); **bei 200 ml/m^3^**: Muttertiere: KG-Zunahme GD 6–21 ↓ (–47 % im Vgl. zur Kontrolle), abs. u. rel. Milzgewicht ↑, Totalresorptionen/trächtige Tiere ↑ (0/23, 0/22, 1/22, 0/23, 9/25*); Feten: Zahl nicht lebensfähiger Implantationen/Wurf ↑ (0,5 ± 0,7; 0,3 ± 0,5; 0,2 ± 0,4; 0,4 ± 1,1; 4,0 ± 4,6*), Resorptionen/Wurf ↑ (0,0 ± 0,0; 0,0 ± 0,0; 0,1 ± 0,3; 0,0 ± 0,0; 2,8 ± 4,7*), Anteil lebender Feten/Wurf ↓ (95,7 ± 5,9; 97,5 ± 4,3; 93,3 ± 21,4; 94,4 ± 12,9; 52,7 ± 45,1*), Ossifikationsverzögerungen; **keine Teratogenität**	Greim 2007; Tyl et al. 1984
**Kaninchen**, Neuseeländer, 24 ♀	**ähnlich OECD TG 414**, **GD 6–18**, 0, 25, 50, 100, 200 ml/m^3^, Ganzkörperexposition, 6 h/d, Untersuchung GD 29	**100 ml/m^3^: NOAEC Entwicklungs- u. Maternaltoxizität**; **ab 200 ml/m^3^**: Muttertiere: Mortalität ↑ (4/24, nicht statistisch sign.), KG ↓ (GD15: ca. 10 %; GD21: ca. 12 %; GD29: ca. 9 %), KG-Zunahme GD 6–21 ↓ (74,2 ± 233,4; 67,8 ± 202,5; 92,4 ± 146,4; 28,3 ± 247,9; –11,0 ± 152,3 g); Gewicht trächtiger Uterus ↓, Aborte ↑ (4/24, nicht statistisch sign.); Feten: Gesamtzahl Implantationen/Wurf ↓ (9,8 ± 3,2; 9,5 ± 2,3; 9,2 ± 2,7; 9,0 ± 3,3; 8,4 ± 1,9*), Anzahl lebensfähige Implantationen/Wurf ↓ (9,0 ± 2,9; 8,4 ± 3,0; 8,5 ± 2,2; 7,6 ± 3,4; 7,2 ± 2,4*), Corpora lutea ↓ (11,7 ± 2,1; 10,8 ± 2,9; 10,5 ± 2,5; 11,3 ± 2,7; 10,1 ± 2,1*); **keine Teratogenität**	Greim 2007; Tyl et al. 1984

^*^p < 0,05

d: Tag; GD: Gestationstag; Nr: Nummer; sign.: signifikant; TG: Test Guideline (Prüfrichtlinie)

In einer pränatalen Entwicklungstoxizitätsstudie ähnlich der OECD-Prüfrichtlinie 414 an F344-Ratten kam es ab 100 ml/m^3^ bei den Feten zu Ossifikationsverzögerungen bei gleichzeitiger Maternaltoxizität, wie reduziertes Körpergewicht, verzögerte Körpergewichtszunahme und hämatologische Effekte, Hämaturie und Hämoglobinurie. Bei 200 ml/m^3^ waren die Anzahl nicht lebensfähiger Implantationen/Wurf und der Resorptionen/Wurf erhöht sowie der Anteil lebender Feten/Wurf erniedrigt. Gleichzeitig trat massive Maternaltoxizität (hämatologische Effekte, reduzierte Körpergewichtszunahme, erhöhte absolute und relative Milzgewichte) auf. Die hämatologischen Effekte waren gekennzeichnet durch eine reduzierte Erythrozytenzahl, erhöhte Hämoglobin- und Hämatokritwerte, eine erhöhte Zellgröße der Erythrozyten und einem erhöhten Quotienten Hämoglobinwert/Zelle. Diese Effekte sind konsistent mit einer Zerstörung der reifen Erythrozyten und einer Freisetzung von unreifen und/oder jungen Erythrozyten in die periphere Zirkulation, was einer hämolytischen Wirkung entspricht (Greim 2007; Tyl et al. 1984). Es ist plausibel, anzunehmen, dass die entwicklungstoxischen Effekte, Ossifikationsverzögerungen und Embryoletalität, fast ausschließlich auf die hämolytische Wirkung der Butoxyessigsäure zurückzuführen sind.

Bei Neuseeländer-Kaninchen traten in einer pränatalen Entwicklungstoxizitätsstudie ähnlich der OECD-Prüfrichtlinie 414 bei der höchsten Konzentration von 200 ml/m^3^ eine statistisch signifikant erniedrigte Anzahl von Corpora lutea, eine verringerte Gesamtzahl von Implantationen und Anzahl lebensfähiger Implantationen auf. Gleichzeitig kam es zu erheblicher Maternaltoxizität, wie vermindertes Körpergewicht, sogar mit Körpergewichtsverlust, Mortalität und Aborten. Hämolytische Effekte wurden nicht festgestellt (Greim 2007; Tyl et al. 1984). Die verringerte Gesamtzahl von Implantationen sowie die erniedrigte Anzahl lebensfähiger Implantationen sind mit einer reduzierten Anzahl Corpora lutea zu erklären. Letzterer Effekt ist nicht als entwicklungstoxischer Effekt anzusehen. Für die Maternaltoxizität ist eine steile Konzentrations-Wirkungs-Beziehung gegeben.

## Bewertung

Das grundlegende Vorgehen zur Bewertung eines Arbeitsstoffes ist der MAK- und BAT-Werte-Liste zu entnehmen (DFG 2025).

Kritische Effekte sind die sensorische Reizwirkung nach Inhalation beim Menschen und die hämatotoxische Wirkung bei der Ratte.

**Fruchtschädigende Wirkung. **In Entwicklungstoxizitätsstudien an Ratte, Maus oder Kaninchen treten nach inhalativer bzw. oraler Verabreichung bei maternaltoxischen Konzentrationen entwicklungstoxische Effekte auf. Beobachtet wurden vor allem erhöhte Mortalität und Resorptionen von Feten bzw. Embryonen sowie verzögerte Ossifikationen (ECETOC 2005 b; Fastier et al. 2005; Greim 2007). Als NOAEC/NOAEL für Entwicklungstoxizität nach Inhalation lassen sich bei Ratten 50 ml/m^3^ und bei Kaninchen 100 ml/m^3^ (Greim 2007; Tyl et al. 1984) sowie nach oraler Gabe bei Ratten 100 mg/kg KG und Tag (Greim 2007) und bei Mäusen 650 mg/kg KG und Tag (Greim 2007; Wier et al. 1987) ableiten. Die entsprechenden Effektkonzentrationen liegen nach Inhalation bei Ratten bei 100 ml/m^3^ und bei Kaninchen bei 200 ml/m^3^ (Greim 2007; Tyl et al. 1984) sowie nach oraler Gabe bei Ratten bei 200 mg/kg KG und Tag (Greim 2007) und bei Mäusen bei 1000 mg/kg KG und Tag (Greim 2007; Wier et al. 1987). Teratogene Effekte wurden bei Ratten, Mäusen und Kaninchen nicht festgestellt (ECETOC 2005 a, b; Fastier et al. 2005; Greim 2007).

Zur Bewertung werden die Inhalationsstudien herangezogen, weil sie der Arbeitsplatzexposition besser entsprechen als die oralen Studien. Die errechneten Abstände zwischen den NOAEC für Entwicklungstoxizität von 50 ml/m^3^ (Ratte) bzw. 100 ml/m^3^ (Kaninchen) und dem MAK-Wert unter Berücksichtigung des erhöhten Atemvolumens (1:2) betragen für Ratten das 2,5-Fache und für Kaninchen das 5-Fache des MAK-Wertes von 10 ml/m^3^.

Bei den Kaninchen ist eine direkte entwicklungstoxische Wirkung aufgrund im Vordergrund stehender Maternaltoxizität, die jedoch nicht durch Hämolyse verursacht wird, nicht anzunehmen.

Bei der Ratte kommt es ab 100 ml/m^3^ zu Ossifikationsverzögerungen und Embryoletalität bei gleichzeitiger Maternaltoxizität, wie reduziertes Körpergewicht, verzögerte Körpergewichtszunahme und hämolytische Effekte (Greim 2007; Tyl et al. 1984). Es ist plausibel, anzunehmen, dass diese entwicklungstoxischen Effekte fast ausschließlich auf die hämolytische Wirkung der Butoxyessigsäure zurückzuführen sind. Damit kommt zum 2,5-fachen Abstand zwischen der NOAEC für Entwicklungstoxizität und dem MAK-Wert zusätzlich die deutlich geringere Empfindlichkeit der menschlichen Erythrozyten hinzu, also ein toxikodynamischer Unterschied der etwa den Faktor 16 beträgt. Damit ergibt sich ein ausreichender 40-facher Abstand (2,5 × 16) zwischen der NOAEC für Entwicklungstoxizität und dem MAK-Wert.

Daher wird für den MAK-Wert von 2-Butoxyethanol in der Höhe von 10 ml/m^3^ die Zuordnung zur Schwangerschaftsgruppe C bestätigt.
